# Reconstructing phosphorylation signalling networks from quantitative phosphoproteomic data

**DOI:** 10.1042/EBC20180019

**Published:** 2018-08-02

**Authors:** Brandon M. Invergo, Pedro Beltrao

**Affiliations:** European Molecular Biology Laboratory, European Bioinformatics Institute (EMBL-EBI), Wellcome Genome Campus, Cambridge CB10 1SD, U.K.

**Keywords:** biological networks, intracellular signaling, phosphorylation/dephosphorylation, systems biology

## Abstract

Cascades of phosphorylation between protein kinases comprise a core mechanism in the integration and propagation of intracellular signals. Although we have accumulated a wealth of knowledge around some such pathways, this is subject to study biases and much remains to be uncovered. Phosphoproteomics, the identification and quantification of phosphorylated proteins on a proteomic scale, provides a high-throughput means of interrogating the state of intracellular phosphorylation, both at the pathway level and at the whole-cell level. In this review, we discuss methods for using human quantitative phosphoproteomic data to reconstruct the underlying signalling networks that generated it. We address several challenges imposed by the data on such analyses and we consider promising advances towards reconstructing unbiased, kinome-scale signalling networks.

## Introduction

Cells transduce and integrate environmental stimuli through a variety of intracellular signalling mechanisms in order to generate appropriate and timely physiological responses. The reversible phosphorylation of proteins by protein kinases is a primary means of modulating the activities of substrate proteins in response to a stimulus. As protein kinases themselves are often regulated by phosphorylation, cascades of phosphorylation events on protein kinases form the backbone of many intracellular signalling pathways. The accumulation of biochemical knowledge of such events has allowed the reconstruction of many of these pathways, which has opened the possibility to study and to model signalling processes at the systems level. This has provided the unprecedented ability to predict cellular outcomes from the phosphorylation state of a pathway following perturbation [1–4]. However, much remains to be discovered about the composition of and cross-talk between these pathways.

## The current state of signalling pathway annotation

Several databases map the networks of regulatory relationships between signalling proteins. Some, such as KEGG [5] or Reactome [6,7], are pathway-focused, compartmentalizing reactions according to the biological functions in which they operate. Others, like SIGNOR [8] or PhosphoSitePlus [9], do not necessarily distinguish pathways and, therefore, allow the construction of large-scale, directed networks of known regulatory relationships. Such databases depend on the curation of knowledge from literature, albeit with different methods or criteria. They also have different release cycles, with some seeing regular updates while others have not been kept up-to-date with the latest publications and data. As a result, there is a notable lack of overlap between them and inconsistencies have been noted for the direction or regulatory sign (stimulation or inhibition) of relationships [10]. Efforts have been made to compile the information contained in these disparate resources to arrive at a consensus protein signalling network, such as OmniPath, which ultimately includes relationships for approximately 39% of the proteome [10].

**Figure 1:**
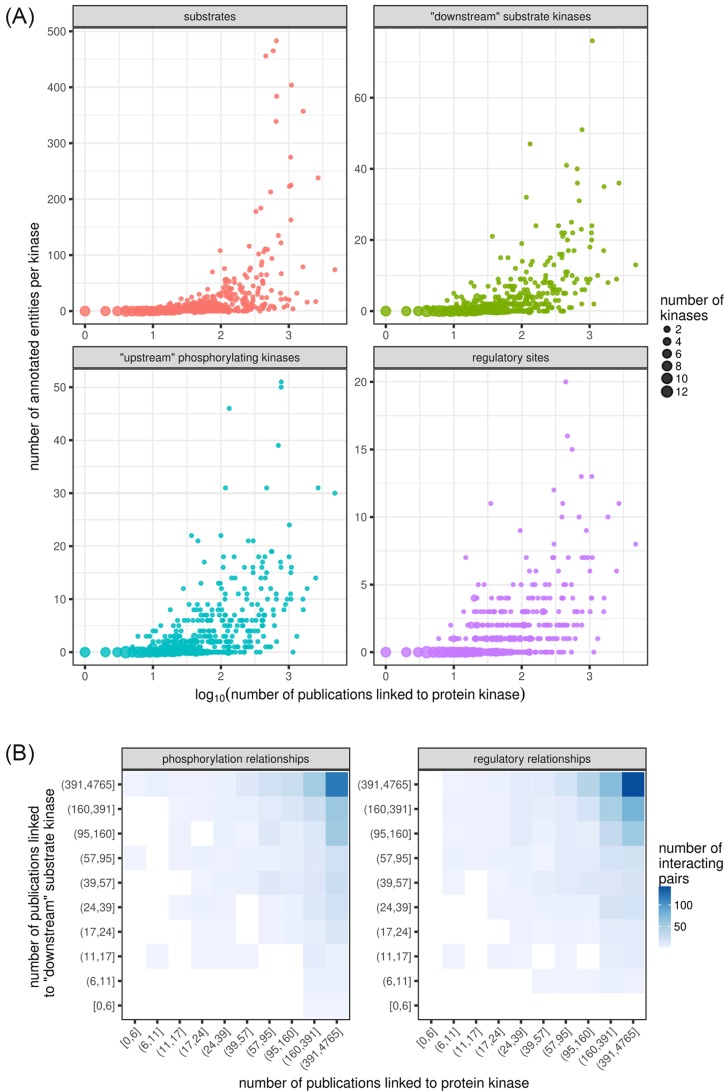
Study bias is a hindrance to comprehensive signalling pathway reconstruction (**A**) Well-studied human protein kinases may appear to be signalling hubs. They have more annotated substrates (top-left panel), including more substrates that are other protein kinases (top-right). They also are annotated to be substrates of more protein kinases themselves (bottom-left) and to carry more activity-regulating phosphorylation sites (bottom-right). Linked publications were retrieved from NCBI Entrez Gene database for each human protein kinase and filtered to remove publications associated with more than ten kinases [11]. Substrate and regulatory site counts were retrieved from PhosphoSitePlus [9]. All data were retrieved on 1 May 2018. (**B**) Literature-derived signalling networks are biased towards canonical pathways: annotated kinase-kinase phosphorylation (left) and regulatory (right) relationships tend to be between well-studied kinases, while relationships involving lesser studied kinases remain unknown. Publication counts for each kinase were binned into ten categories (deciles) of approximately 50 protein kinases each. Human kinase-kinase regulatory relationships were retrieved from OmniPath [10], restricting to directed relationships with support from at least two source databases. Autophosphorylation and autoregulation were omitted for clarity.

Nevertheless, the curation of signalling pathway information from studies produced by diverse research groups suffers from the problem of study bias, in which proteins with known function receive more research attention. In this way, a well-studied protein kinase will have more known substrates or better understood regulatory mechanisms, potentially appearing to be a signalling hub (Figure 1A). Its activity will thus be over-represented in literature-based pathway reconstructions (Figure 1B). As a result, the core events comprising a few canonical pathways become well-resolved, while a large space of the potential, kinome-wide phosphorylation signalling network remains largely unexplored (Figure 1B, right panel). This pattern, in turn, leads to formulating and testing new hypotheses within the framework of these known pathways, exacerbating the bias.

For example, the human proto-oncogenic tyrosine-protein kinase, Src (encoded by the *SRC* gene) is a common research target: as of 1 May 2018, it is linked on Entrez Gene [11] to 1096 PubMed publications (see Figure 1 for more information). It is annotated in PhosphoSitePlus [9] to phosphorylate 76 other kinases, the highest number among human kinases. Notably, the other, less well-studied Src-family kinases have significantly fewer: e.g. Fyn (FYN; 428 publications) is annotated to phosphorylate 22 other kinases; Lck (LCK; 395 publications), 16; and Yes (YES1; 78 publications), only 3. Although human protein-kinase signalling has been studied for decades, 262 kinases presently lack annotations for any ‘downstream’ substrates that are protein kinases, and 171 have no annotations for protein substrates whatsoever. These should of course not be considered as peripheral to intracellular signalling: serine/threonine-protein kinase SBK1 (3 publications), for example, lacks any annotations for known substrates, upstream kinases or regulatory phosphosites, yet it is broadly expressed and is dysregulated in multiple cancers [12].

This is not to imply that increased research interest in these kinases will necessarily result in more substrate annotations. Lck, for example may ultimately be a far more specific kinase than Src. In particular, highly tissue-specific protein kinases such as retinal photoreceptor cell-specific G-protein-coupled receptor kinases 1 and 7 (*GRK1* and *GRK7*) can be expected to have relatively few physiological substrates. Similarly, not all protein kinases are expected to be regulated by phosphorylation, so it is not surprising that some well-studied kinases lack annotations for regulatory sites or upstream kinases (Figure 1A, bottom panels). However, it does suggest that caution must be exercised when drawing network-level conclusions about protein kinases from literature-derived pathways, as this bias obfuscates any results obtained by graph-theoretic (i.e. shortest path, centrality or clustering analyses) or pathway modelling approaches.

It is likely that despite intensive efforts and decades of research, a large fraction of the human protein-kinase signalling network remains to be discovered. The fully resolved network would provide a more accurate perspective of the systems level contexts of signalling events and the roles played by individual protein kinases. Thus, we require means of performing unbiased pathway reconstruction in order to accelerate the discovery process and to gain a more comprehensive view of intracellular signalling.

## A brief survey of signalling pathway reconstruction methods for quantitative phosphorylation data

In order to reconstruct the intracellular signalling network in an unbiased manner, we must turn to high-throughput data-generation methods for the inference of regulatory signalling relationships. For quantifying the phosphorylation state of proteins, immunodetection via phospho-specific antibodies is an established and often-used method. However this approach limits the number of proteins that can be included in a given study. More recently, phosphoproteomics, the identification and quantification of phosphorylated proteins on a proteomic scale via LC-MS/MS, has emerged as a common technique (phosphoproteomics has been widely reviewed elsewhere; see e.g. [13]). Importantly, it allows the quantification of phosphorylation state on thousands of proteins in a single experiment, comprising a large proportion of the instantaneous intracellular signalling state.

Efforts have been made to exploit this quantitative phosphorylation-state data in order to reconstruct the underlying signalling pathways that generated it. These have included two formal competitions under the Dialogue for Reverse Engineering Assessment and Methods (DREAM) challenge framework, which together attracted hundreds of submissions [14,15]. Given the wealth of methods collected within even these two contexts, it would not be feasible to enumerate here all the options available for performing signalling pathway reconstruction. Nevertheless, we highlight a few of the broader methodological trends.

## Methods for steady-state data

Perhaps the most common means of collecting phosphorylation data involves measuring at a fixed time point. Inferences about the dynamic network topology can then be made from associations of protein kinase activities measured across different conditions, such as multiple cell lines, stimuli or perturbation strategies. This would entail measuring kinase activity across a large panel of conditions with, for example phospho-specific antibodies targeting known kinase regulatory sites. If two kinases change in activity in a correlated manner, it would be possible to infer that one may regulate the other. Although correlative or information content-based methods can detect such associations across sufficient conditions, the resulting networks are undirected, failing to identify the regulator compared with the substrate, and often suffer from the inability to discern direct from indirect associations. However, more sophisticated methods have been produced that utilize information imposed by experimental design to produce directed regulatory networks.

Modular response analysis (MRA), an early effort in this regard, takes advantage of successive and exhaustive perturbations of each of the known components in a pathway [16,17]. This approach linearly approximates how the activity of each component changes given perturbations in each of the other components. This results in signed coefficients connecting each kinase in the network, reflecting inhibition, attenuation or amplification of the signal as it passes from one kinase to the next. By extending the experimental design to measure the full perturbation scheme under different conditions, context-specific networks can be derived. For example, Santos et al. [18] investigated the mitogen-activated protein kinase (MAPK) signalling network in PC-12 cells under epidermal or neuronal growth factor (EGF or NGF) stimulation. In addition to recovering the canonical regulatory relationships within the pathway, MRA also revealed distinct, context-specific modes of feedback under these two stimuli: feedback from MAPKs to Raf kinases is negative under EGF stimulation and positive for NGF stimulation.

A practical challenge for applying MRA at larger scales is the ability to design and execute a sufficient perturbation scheme with increasing numbers of protein kinases under study. Two MRA variants have been proposed that circumvent this limitation. In the first, a maximum likelihood-based approach is employed to optimize the estimated network coefficients from the measured responses, even in the absence of perturbations to all kinases [19,20]. The second approach reformulates the task of the estimation of network coefficients as a linear regression for each kinase. A Bayesian model-variable selection method is then used to estimate the coefficients of the kinase’s incoming regulatory edges [21,22].

Indeed, by explicitly modelling uncertainty and integrating prior probabilistic knowledge, Bayesian statistics provide a powerful paradigm to approach the problem of signalling network reconstruction. For example, Oates et al. [23] modelled phosphoprotein state with a non-linear function reflecting biochemical equilibrium kinetics of the regulating kinases. They then inferred the network of regulatory relationships in a Bayesian framework by finding the network topology that provided the most likely models of the phosphoprotein states given the data. This non-linear approach significantly improved the predictive performance on simulated data produced by a model of MAPK signalling compared with a linear formulation similar to [21] and non-Bayesian linear methods. Unlike MRA, this method has no strict requirement for a full array of perturbations (indeed, it was successfully applied in [23] to an unperturbed phosphoprotein dataset from a panel of breast cancer cell lines).

## Methods for time-resolved data

Given the dynamic nature of intracellular signalling, the assumptions imposed by the above methods render them inappropriate for cases where a steady state is not reached under physiological conditions. Increasingly, time courses of phosphorylation are measured in order to capture such dynamics. Accordingly, new methods have been developed to take advantage of the resulting temporal dependencies between samples.

A theoretical foundation for reconstructing networks from time-resolved data is laid out by Oates and Mukherjee [24], closely resembling the Bayesian variable selection for linear regression later employed in the reformulation of MRA. Perhaps the most important practical result of their work was the demonstration that evenly sampled time courses provide far stronger performance than the unequal sampling used in many biological experiments. Bayesian variable selection can also be used in the context of dynamic Bayesian networks (DBNs) [25,26]. This method was applied to a time-course of phosphorylation in a breast cancer cell line at eight time points and under four growth conditions. It successfully recapitulated previously described relationships while also producing novel predictions, such as a validated phosphoregulation of signal transducer and activator of transcription 3 (STAT3) by MAPK, reflecting a novel mode of cross-talk between MAPK and JAK/STAT signalling [25]. In a larger experiment, DBNs were also successful in inferring context-specific signalling networks in four breast cancer cell lines under eight stimulus conditions [26]. Finally, the biochemical kinetics approach in [23] has since been extended into a general framework for the inference of signalling pathways and dynamics from phosphorylation time-course data, which appears to out-perform DBNs [27].

A fundamentally different strategy to the ones described above lies in building logic models based on prior qualitative knowledge and then optimizing them to fit experimental data [28]. In this case, the data are usually considered in the context of discrete time and are normalized to binary measurements. Importantly, the derived topology is not restricted to prior knowledge and the method has been shown to be useful in predicting new regulatory relationships [28]. Furthermore, continuous, ordinary differential equation (ODE)-based models can be recovered from a Boolean logic model, which has been applied to dual time-course phosphoproteomic data in order to analyse cross-talk between the osmotic stress and pheromone response pathways in budding yeast [29,30]. In fact, these methods are not restricted to time-course datasets. For example, Terfve et al. [31] demonstrated logic models as powerful means to reconstruct large-scale signalling pathways on steady-state data. They produced context-specific networks by training logic models to phosphoproteomic data from breast cancer cell lines that were exposed to a panel of kinase inhibitors. Most recently, this method has advanced towards unbiased network reconstruction, through the introduction of a method to infer a purely data-driven prior network [32].

## Challenges inherent to pathway reconstruction from phosphoproteomic data

Although the ability to probe the phosphorylation signalling state at a proteomic scale holds great promise, presently we face several challenges in producing large-scale maps of intracellular signalling. Perhaps the most significant constraint is the combinatorial explosion of possible network topologies as more protein kinases are taken under consideration. On the one hand, exhaustively testing the space of all possible networks, or even thoroughly sampling it, quickly becomes computationally infeasible. On the other hand, as the number of variables under consideration grows, so too should the number of independent measurements be increased. Concerning the latter difficulty, although phosphoproteomics experiments are high-throughput with regards to data content, they must still be considered low-throughput in producing multiple, independently analysed samples. As a result, a typical experiment presently will have fewer than ten conditions, whereas hundreds, if not thousands, of conditions would be desirable to confidently discern regulatory relationships between hundreds of protein kinases. A recent compilation of data from nearly 400 human quantitative phosphoproteomics experiments may help to overcome this problem [33].

Technical limitations of LC-MS/MS pose other difficulties in network reconstruction. For example, one must enzymatically digest proteins into peptides prior to analysis. As a result, a given phosphosite of interest may appear on several peptides due to alternative cleavage sites or multiply phosphorylated forms of the peptide. Thus, discerning the state of a single phosphorylation site may require combining several pieces of evidence. A more serious challenge imposed by LC-MS/MS is the relative sparseness of the resulting data. Standard shotgun proteomics techniques result in low reproducibility of phosphopeptide identification due to stochastic sampling, particularly affecting low-abundance peptides [34]. Within a single experiment, reproducibility across samples can be improved by employing multiplexed peptide-labelling techniques such as tandem mass tag (TMT) labelling. However the specific peptides identified in each multiplex experiment will be still be stochastic, limiting overlap between experiments. As a result, relying on a specific phosphopeptide or set of phosphopeptides as reliable markers of protein-kinase activity across a large number of conditions proves to be difficult.

Recent developments in targeted LC-MS/MS technology may open new avenues for efficient and reproducible quantification of phosphopeptides [35]. In general, these methods first require phosphopeptide spectra to be identified using shotgun proteomics via data-dependent acquisition (DDA). They then use this spectral database to identify targeted phosphopeptides in subsequent analyses. These methods provide higher reproducibility and sensitivity of peptide detection as well as improved dynamic range of quantification. If specific pathways are of interest, parallel reaction monitoring (PRM) provides highly reproducible and sensitive quantification of a set of phosphopeptides [36,37]. For example, PRM has been applied to a set of 96 phosphopeptide probes and achieved detection reproducibility of 95% across approximately 200 samples [38]. Recently developed data-independent acquisition methods (DIA) offer the potential of performing truly high-throughput targeted analyses [39,40]. However, owing to the complexity of the spectra produced following DIA, the identification of the peptide precursors comprising them remains a challenging computational problem, particularly in deconvoluting the contribution of phosphorylated forms of peptides. Although this remains an open area of research, recent developments in analysis methods show promising improvements over traditional DDA approaches [41,42].

## Inference of kinase activity from quantitative phosphorylation data

In order to reconstruct a signalling pathway from quantitative phosphorylation data, protein kinase activities must be inferred indirectly from phosphorylation state. One way of achieving this is by quantifying changes at a regulatory phosphorylation site on the protein kinase. Caution must be used in this case, as phosphorylation might induce or inhibit activity. Thus, anti-correlation of regulatory phosphorylation states between two kinases might reflect an activating or an inhibitory relationship, depending on the functions of the regulatory sites. Furthermore, some protein kinases require multiple phosphorylations to be fully activated, which must be measured together in order to capture the full activation state of the kinase. In practice, however, the ability to apply this method is limited by available knowledge: activity-regulating phosphosites are reported in PhosphoSitePlus for only 265 of the 508 human protein kinases annotated in UniProt [9]. Alternatively, kinase activity can be inferred by tracking the state of a known substrate of the kinase. However, redundancy in sequence specificity exists within families of kinases, hindering the confident identification of unique substrate sites. For example, while PhosphoSitePlus reports substrates for 333 human protein kinases, only 284 have uniquely identified substrates. Given the study bias illustrated in Figure 1, other less well-studied kinases may indeed also phosphorylate these ‘unique’ substrates. The emergence of high-throughput methods to discover protein kinase substrates, such as *in vivo* expression of ATP analog-sensitive variants of kinases [43,44] or *in vitro* assays of kinase reactions on dephosphorylated cellular proteins [45], promises a means to rapidly identify unique substrates for a broader set of protein kinases than presently available.

Dependence on a single phosphorylation site, be it a regulatory one or a substrate site, is risky given the stochasticity of phosphopeptide detection in shotgun phosphoproteomics. One way around this is to estimate protein kinase activity from multiple substrate phosphosites, such that in any given experiment or condition, some subset of them has been quantified. One such method estimates a protein kinase’s activity from the phosphorylation state of its neighbours on a literature-derived protein–protein interaction (PPI) network [46]. The authors of this method applied a network-diffusion technique to these inferred activities to reconstruct signalling pathways on the PPI network. An alternative, kinase-substrate enrichment analysis (KSEA), depends on annotated kinase-substrate relationships [47,48]. This method has been used to investigate the relationships of protein kinase activities in phosphoproteomic data measured from breast cancer cell lines under a panel of protein kinase inhibitors [49]. On a larger scale, Ochoa et al. [33] employed KSEA to compare and relate the overall intracellular signalling states across hundreds of conditions. Nevertheless, neither of these experiments employed KSEA specifically for the task of signalling pathway reconstruction, which remains an intriguing application.

## Towards an unbiased, kinome-scale phosphorylation signalling network

**Figure 2:**
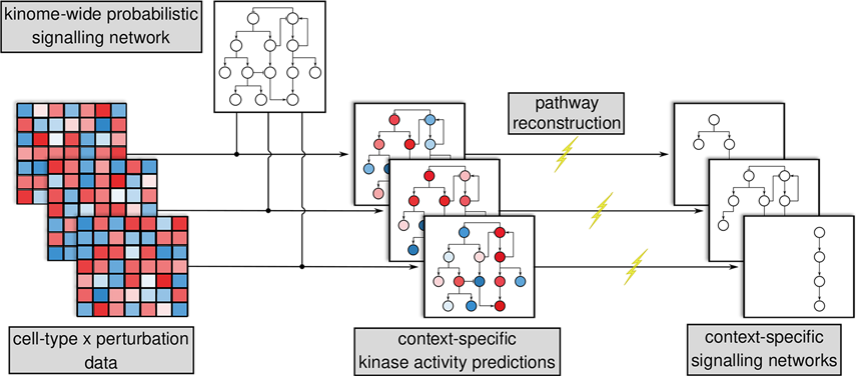
A kinome-wide, probabilistic regulatory network would serve as a foundation upon which to derive context-specific signalling pathways Phosphoproteomic and other data are collected from large-scale experiments in which different cellular contexts (e.g. cell lines, tissue types or stimulus regimes) are measured across several perturbations and/or time points. Kinase activities are inferred from these data and mapped on to the probabilistic network. Further network refinement is then performed to reconstruct context-specific signalling pathways.

Although phosphoproteomics promises the ability to measure the entirety of the cellular signalling state, the majority of phosphorylation-based signalling network reconstruction efforts to date have focused on the analysis of specific pathways and, therefore, have been subjected to the study bias inherent in pathway annotations. As described above, this limitation can largely be attributed to practical challenges in both data collection and computational analysis. However, a full-kinome regulatory network reconstruction would be valuable, particularly in identifying regulation of lesser studied pathways or in mapping complex cross-talk between pathways. It would also form an unbiased foundation upon which new data can be interpreted in order to elucidate, in detail, specific signalling responses (Figure 2).

Given that signalling can be highly context specific, this should take the form of either a probabilistic network, in which an edge reflects some estimated probability that one kinase regulates another or an executable model, in which context specificity can be simulated by modulating the model inputs. Given the number of parameters required for such a model and the amount of data necessary to confidently fit the parameters, the probabilistic network is currently a more feasible option (however, progress is being made in parameter-estimation techniques for large systems; see, e.g. [50]). Context-specific probabilities can be derived by integrating protein abundance data. They can then be validated against phosphoproteomic data generated after specific kinase inhibition in order to produce internally consistent networks [51]. Such context-specific probabilistic networks would be particularly useful as unbiased, data-driven priors for pathway-level network reconstruction [52].

We cannot yet say what the basic characteristics of this kinome-scale network should be. In particular, although kinase substrate-specificity implies that the network should be sparse, any sparsity restrictions that we impose on the network presently would be arbitrary. Confident substrate sequence-specificity profiles can only be produced for a fraction of the human kinome, and these display degeneracy at most positions and redundancy among themselves, resulting in a dense network of kinase-substrate predictions. Regardless, depending on observed or predicted substrates is not sufficient to infer regulatory network sparsity, as some phosphorylation sites may not be functional [53–55]. Based on current knowledge, most protein kinases are not promiscuous in regulating others kinases; that is, they induce a scale-free network of regulatory relationships, with only a few highly connected nodes [10,56]. However, it is difficult to distinguish whether this observed pattern is due to actual biological constraint, as is generally supposed, or study bias resulting in the identification of more regulatory relationships for a few well-studied protein kinases (Figure 1). Thus, the true sparsity of the network remains unknown.

Assuming that the real network is, indeed, sparse, data-driven network reconstruction methods will have to contend with a space of false regulatory relationships that dwarfs the space of true relationships. Although the goal is to detect previously unknown regulatory relationships in addition to recovering known ones, network sparsity implies that these will be exceedingly rare. Given the currently low dynamic range of quantitative shotgun proteomics techniques, it is likely that current network reconstruction methods are not sensitive enough to capture salient relationships without also introducing overwhelming numbers of false-positives. However, the higher dynamic ranges achievable with targeted proteomics techniques may help to improve performance.

Although several challenges face such an endeavour, achieving a human kinome-scale signalling network will provide an unprecedented understanding of how cells integrate information across their multiple, interconnected pathways under different conditions. By mapping new phosphoproteomic, proteomic or transcriptomic data on to it, context-specific subnetworks could be derived, studied and validated (Figure 2). In this way, it would be a sound replacement for the bias-laden literature-derived networks currently in use for pathway-level network reconstructions. This will, in turn, feed back into our detailed understanding of individual pathways through improved models of signalling dynamics and behaviour.

## Summary

- Our current knowledge of protein kinase-based signalling is biased towards well-studied kinases.
- Proteomic-scale quantitative phosphorylation data can be exploited to reconstruct the signalling network that generated it in an unbiased manner.
- Several methods for network reconstruction exist, however experimental and computational challenges hinder their application to large-scale networks.
- There is a need for unbiased, kinome-wide signalling network reconstruction to support and accelerate the discovery of new signalling pathways and the refinement of known ones.

## Abbreviations

DBN: dynamic Bayesian network
DDA: data-dependent acquisition
DIA: data-independent acquisition
EGF: epidermal growth factor
KSEA: kinase-substrate enrichment analysis
MAPK: mitogen-activated protein kinase
MRA: modular response analysis
PPI: protein–protein interaction
PRM: parallel reaction monitoring
